# “Cutaneous Rabbit” Hops Toward a Light: Unimodal and Cross-Modal Causality on the Skin

**DOI:** 10.3389/fpsyg.2012.00427

**Published:** 2012-10-22

**Authors:** Tomohisa Asai, Noriaki Kanayama

**Affiliations:** ^1^Department of Psychology, Chiba UniversityChiba, Japan; ^2^Department of Cognitive and Behavioral Science, Graduate School of Arts and Sciences, The University of TokyoTokyo, Japan

**Keywords:** cutaneous rabbit effect, multi-modal integration, vision, tactile, localization

## Abstract

Our somatosensory system deals with not only spatial but also temporal imprecision, resulting in characteristic spatiotemporal illusions. Repeated rapid stimulation at the wrist, then near the elbow, can create the illusion of touch at intervening locations along the arm (as if a rabbit is hopping along the arm). This is known as the “cutaneous rabbit effect” (CRE). Previous studies have suggested that the CRE involves not only an intrinsic somatotopic representation but also the representation of an extended body schema that includes causality or animacy perception upon the skin. On the other hand, unlike other multi-modal causality couplings, it is possible that the CRE is not affected by concurrent auditory temporal information. The present study examined the effect of a simple visual flash on the CRE, which has both temporal and spatial information. Here, stronger cross-modal causality or correspondence could be provided. We presented three successive tactile stimuli on the inside of a participant’s left arm. Stimuli were presented on the wrist, elbow, and midway between the two. Results from our five experimental manipulations suggest that a one-shot flash enhances or attenuates the CRE depending on its congruency with cutaneous rabbit saltation. Our results reflect that (1) our brain interprets successive stimuli on the skin as motion in terms of time and space (unimodal causality) and that (2) the concurrent signals from other modalities provide clues for creating unified representations of this external motion (multi-modal causality) as to the extent that “spatiotemporal” synchronicity among modalities is provided.

## Introduction

Our daily lives are rich with information from the physical world. While some limits are imposed by sensorineural imprecision (for a review, see Knill and Richards, 1996), the brain has developed strategies to deal with these limitations, including the utilization of prior knowledge and integration among multi-modal information. A percept that misrepresents physical reality (i.e., an illusion) is both a consequence of and a clue as to the brain’s expectations regarding the external world (Goldreich, 2007).

The brain takes advantage of prior knowledge to enhance its perceptual resolution. In the case of tactile perception, spatial imprecision due to low receptor density poses a particular challenge (Goldreich, 2007). Even without the benefit of exploratory movements, the fingertips’ resolving power – the most discriminating tactile sensor among primates – is on the order of 1 mm (Weinstein, 1968; Johnson and Phillips, 1981). However, the forearm has less acuity: it resolves detail on the order of 1 cm (Weinstein, 1968). This is the case even though the brain contains a representation of the body map in the primary somatosensory cortex (S1; Penfield and Boldrey, 1937) which reflects the locations of physical stimuli on the skin. Furthermore, given the several-ms jitter in the stimulus-evoked – first-spike latencies of somatosensory cortical neurons (Foffani et al., 2004), the somatosensory system has not only spatial but also temporal imprecision; this results in characteristic spatiotemporal illusions. The “cutaneous rabbit effect” (CRE) might be the best-known of these illusions (Goldreich, 2007). The CRE is a subset of a larger class of tactile saltation illusions elicited when a mechanical stimulus is followed by similar stimuli rapidly applied at nearby locations (Geldard and Sherrick, 1972; Warren et al., 2010). For example, repeated, rapid stimulation at the wrist and then near the elbow can create the illusion of touch at intervening locations along the arm, as if a rabbit is hopping along the arm. The apparent location of each stimulus moves from the actual stimulation site toward the other stimulation sites in a predictable manner depending on factors such as stimulus location and frequency (e.g., Geldard and Sherrick, 1972; Kilgard and Merzenich, 1995; Cholewiak, 1999; Flach and Haggard, 2006).

The CRE is apparently related to the classic tau effect (Goldreich, 2007), in which the more rapidly traversed of two equal distances defined by three stimuli is perceived as being shorter (Helson, 1930). When stimulus timing is held constant, the perceived distance between two stimuli both underestimates and grows in proportion with the actual inter-stimulus distance (Marks et al., 1982; Cholewiak, 1999). In contrast, the kappa effect describes the elevated perceived time between stimuli dilations as the distance between stimuli is increased (Suto, 1952). These effects have been explained on the basis of the hypothetical idea that the sensory system imputes uniform motion to discontinuous dynamic displays; therefore, there is an assumption of constant velocity motion (Jones and Huang, 1982). Also, a recent Bayesian perceptual model replicated the CRE by assuming that the brain expects tactile stimuli to move slowly (Goldreich, 2007) since we have evolved to detect the movement of external agents (Leslie, 1995). The inference that signals have a common underlying cause (in this case, movement) enables us to perceive uniform motion; this is an expression of unimodal causality perception in terms of time and space. A similar argument has been proposed to explain visual motion perception. Certain simple visual displays consisting of moving, 2-D, geometric shapes can give rise to percepts with high-level properties, such as causality and animacy. This suggests that just as the visual system works to recover the physical structure of the world by inferring properties such as 3-D shapes, it also works to recover the causal and social structures of the world by inferring properties such as causality and animacy (Scholl and Tremoulet, 2000).

Multi-modal integration can also assist in circumventing the limits imposed by sensorineural imprecision within each modality. Given that many natural events can be perceived via multiple sensory modalities, we typically have access to multiple features of those events across different senses (Vroomen and Keetels, 2010). It is generally assumed that signals that are congruent among modalities create stronger experiences and richer representations of the world than unimodal signals (for a review, see Woods and Newell, 2004). The ability to combine information from multiple sensory modalities into a single, unified percept is a key element of organisms’ abilities to interact with the external world (Stevenson et al., 2011). This process of perceptual fusion – the amalgamation of multiple sensory inputs into a perceptual gestalt – is highly dependent on the temporal synchrony of sensory inputs (Meredith et al., 1987; Bishop and Miller, 2009; Stevenson et al., 2011). The inference that signals have a common underlying cause, and hence merit integration, is often called the “correspondence problem” or “causal inference” (Parise et al., 2012). The combination of cross-modal information by humans is highly consistent with an optimal Bayesian model of causal inference; this phenomenon is known as “cross-modal causality” (Goldreich, 2007; Beierholm et al., 2009; Schutz and Kubovy, 2009). For example, while at the movie theater, we hear voices as coming from the mouths of characters on the screen, not from the actual speakers (i.e., spatial ventriloquism; Jack and Thurlow, 1973; Alais and Burr, 2004). This is because we make causal inferences between vision and audition: “I hear the voice because I, see the character speaking.” Another example of cross-modal causality can be observed in a simple visual display consisting of moving, 2-D, geometric shapes. Observers usually attribute the launching of one object to another object that abruptly stops in front of a target object (Michotte, 1963; for review, see Scholl and Tremoulet, 2000). Interestingly, a sound marking the onset of the target motion significantly increases the impression of causality. This facilitation is likely due to the observer’s intuitive reasoning that audiovisual stimuli comprise parts of a unitary event (i.e., a collision of two objects producing a bouncing sound; Guski and Troje, 2003). It seems that we prefer to perceive just one (or minimal) cause or agent during multi-modal integration to the extent that temporal synchrony among modalities is provided.

Given the idea that we interpret the outer world through our expectations (where prior knowledge and multi-modal integration is helpful), we might assume that unimodal causality perception (like the tau and kappa effects or the CRE) could be modulated under multi-modal presentation; however, this is controversial (e.g., Flach and Haggard, 2006). Indeed, the tactile tau and kappa effects are also susceptible to cross-modal (visual or auditory) influences (Suto, 1952; Russo and Dellantonio, 1989); other combinations are also possible (e.g., the audiovisual tau effect: Kawabe et al., 2008), indicating the incorporation between unimodal and cross-modal causality perception. Conversely, one previous study has suggested that the CRE is not affected by concurrent auditory temporal information (Flach and Haggard, 2006). In that study, three successive taps were presented on a participant’s arm, and the participant localized the second tap. Although three, concurrent auditory tones were presented, no cross-modal interaction within their localization was observed, suggesting that the CRE is the spatiotemporal dynamics of an early, “unimodal” sensory map (Flach and Haggard, 2006). Another study also suggested that the illusory somatosensory percepts caused by the CRE can affect the primary somatosensory cortex at a location corresponding to the illusory percept (Blankenburg et al., 2006). However, another recent study suggested that the CRE could be experienced outside of the body, where it lacks a specific receptive field in S1, indicating that the CRE involves not only intrinsic somatotopic representations but also those of the extended body schema that result from body–object interactions (Miyazaki et al., 2010). In other words, these representations impart expectations regarding the movement of the external agent.

The present study attempted to extend this literature. As far as we know, there is no published paper that has thus far indicated a multi-modal influence on the CRE. We assumed that the CRE could be modulated by cross-modal influence only if concurrent information has enough power to create “causal inferences” among modalities (Parise et al., 2012). In particular, we examined the effect of simple visual flashes on the CRE. The auditory tones used in previous studies have only provided temporal information since the tones were presented through headphones (Flach and Haggard, 2006). A visual flash, however, has both temporal and spatial information, which should elicit cross-modal correspondence between tactile and visual senses in terms of time and space. We hypothesized that a simple flash could modulate the CRE depending on its location of presentation, similar to reports of the tau and kappa effects (Suto, 1952; Russo and Dellantonio, 1989). The expected results should be important when we consider the mechanism of the CRE, as well as causality perception in the outer world. Is the CRE truly a phenomenon limited to early unimodal somatosensation (Flach and Haggard, 2006)? The CRE is a good method for demonstrating the relativity or interdependency of space and time in somatosensation; furthermore, the CRE reflects our expectation of the world (Goldreich, 2007; Miyazaki et al., 2010). If this is the case, the CRE should be susceptible to multi-modal presentation in order to create a unified representation of moving stimuli on the skin to the extent that the “spatiotemporal” synchronicity among modalities is provided (as well as other multi-modal couplings).

The results of our five successive experiments actually suggested a visual effect on the CRE, but the results are more complicated than we hypothesized (see also the experiment-specific introductions). The present study has suggested that unimodal causality perception would be enhanced but might not be attenuated by cross-modal causality. This could indicate that our brain is tuned to detect the movement of an external agent on the skin since an essential, evolutionarily stable feature of brain function is the detection of animate entities for survival (Schultz et al., 2005; Pratt et al., 2010). Furthermore, we argue that sensory events at a certain time point are influenced by future sensory events; this is referred to as “postdictive” sensation (Eagleman and Sejnowski, 2000).

## Materials and Methods

### Participants

All participants were right-handed university students, and none participated in more than one experiment. They were recruited randomly from an introductory psychology class, and written informed consent was obtained from all participants before the experiments were conducted. All participants reported normal or corrected-to-normal vision, hearing, and somatosensation and no neurological abnormalities. The experiment was conducted in accordance with the Declaration of Helsinki.

### Apparatus

**Figure 1:**
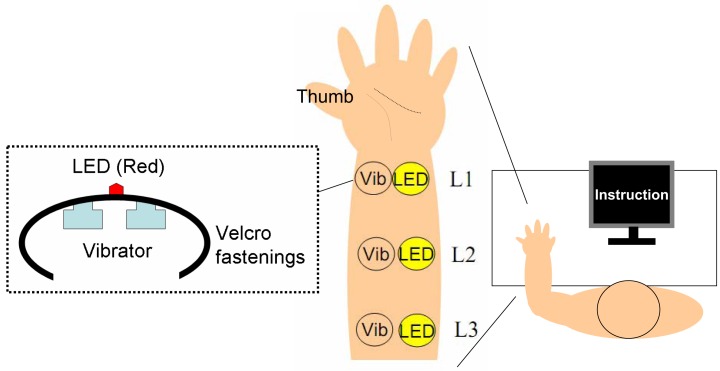
**The experimental apparatus**.

The experiments took place in a silent, dark room. In order to deliver the visual and tactile stimuli, we used a multi-channel signal processor (UA-101, Roland, Shizuoka, Japan) and an amplifier (QuadMic, RME, Haimhausen, Germany) connected to a PC. The tactile stimuli were presented through vibrators (bone conductors: MGD-701, Golden Dance, Osaka, Japan), and visual stimuli were presented using LEDs (3-mm diameter). Two vibrators (10-mm diameter, used to increase the intensity of tactile stimuli) and one red LED were combined using Velcro fastenings onto a band device (see Figure 1). The participants wore three devices on the inside of their left arm: one each at the wrist (Location 1: L1), elbow (L3), and midway (L2) between the two (about 10–13 cm separated each device). The intensities of stimuli (flash and vibrotactile) were set at sufficient levels, and we roughly equalized the subjective intensity of tactile stimuli among the three devices across participants. White noise was presented through a speaker (80-dB SPL) in order to prevent extraneous sounds from influencing the vibrators during the experiment.

### Stimuli

Visual and tactile stimuli were controlled by a sound signal (300 Hz sine wave) using MATLAB (MathWorks, Natick, MA, USA). The stimulus duration was 100 ms, and we presented three successive signals with a 100-ms ISI (inter-stimulus interval). For instance, one sequence was as follows: signal (time 1: *t*1) – blank – signal (*t*2) – blank – signal (*t*3). Thus, the three signals for each trial were presented over a 500-ms duration. The CRE is subject to temporal parameters such as stimulus duration and ISI. According to the results of previous studies (e.g., Blankenburg et al., 2006; Warren et al., 2010) and the results of our own preliminary experiments, these temporal parameters were adjusted so that the typical CRE response (L1–L2–L3 tactile feeling under the L1–L1–L3 tactile stimuli condition) would be observed approximately 50% of the time. This was done because it is necessary to have a margin for the multi-modal interaction (i.e., the effects of visual stimuli on the CRE). The *t*1 and *t*3 signals were identical in all conditions: tactile stimuli for the wrist (L1) at *t*1 and for the elbow (L3) at *t*3. At *t*2, tactile and visual stimuli (though one or the other of these was not present under some conditions) were presented somewhere between L1 and L3, including the midpoint (L2) between the wrist and elbow.

### Procedure

All participants sat in front of the display, and their left arm was supinated on a table (see Figure 1). We instructed participants to relax their left hand during the experiment. Before the experiment began, the participants received a brief training sequence to ensure familiarity with the instruments and experimental requirements. A simple visual and auditory cue signifying the onset of a trial was first presented on the display and through the speaker. Participants then saw their left arm. They were instructed to respond via key press after perceiving a successive visuo-tactile stimulus after a random interval (1000–1500 ms). Although the specific requirements were experiment-dependent, all experiments required participants to report their tactile sensation while ignoring visual stimuli. Participants were informed that they would be presented with three successive stimuli (tactile, flash, or both) per trial, distributed among three devices.

## Experiment 1A

To show that the CRE – an expression of unimodal causality perception – could be modulated by cross-modal influences, we administered a one-shot visual flash accompanied by three successive tactile stimuli. We hypothesized that if the visual flash were presented concurrently with one of the tactile stimuli (and that location were congruent with the CRE saltation), then the CRE would be enhanced. This manipulation would provide causal, spatiotemporal correspondence between the visual (the flash) and tactile (the CRE saltation) senses. We expected that L1–L1–L3 tactile stimuli would be felt as L1–L2–L3 to some extent. Furthermore, a flash on L2 at *t*2 was expected to induce a strengthened L1–L2–L3 tactile sensation in this condition.

### Method

**Figure 2:**
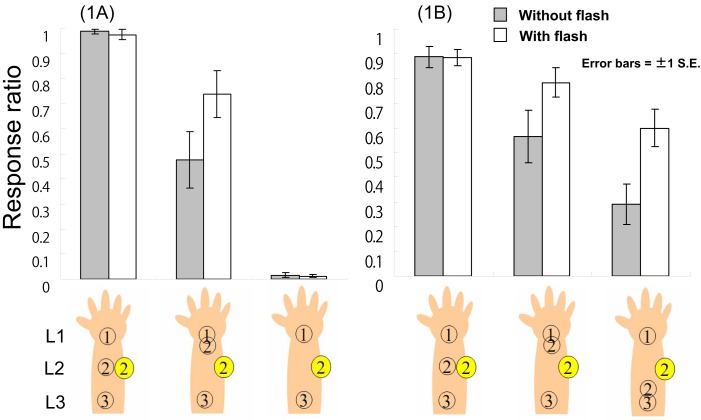
**The response ratio (L1–L2–L3 tactile feeling) in Experiment 1A and B**. Note: The numbers in circles mean the timings ① corresponds to *t*1. For example, the leftmost illustration means that L1 → L2 → L3 tactile stimuli with flash at *t*2 timing.

Twelve university students (four male and eight female, mean age = 18.6 years, range = 18–21) participated in a 3 (patterns of tactile stimuli) × 2 (presence vs. absence of visual stimuli)-factor experiment. For the patterns of tactile stimuli, we presented three successive signals through vibrators: L1–L2–L3, L1–L1–L3, and L1-(blank)-L3. In the visual-stimuli-provided conditions, we presented a one-shot visual signal on the LED located on L2 at *t*2 (Figure 2, left panel). These six conditions were randomly repeated 20 times for each participant. Participants were required to respond (via key press) as quickly as possible using their index fingers after the successive multi-modal stimuli were presented. Participants pressed the right (or left) key immediately when they felt the tactile sensation as L1–L2–L3, regardless of visual stimuli. Participants immediately pressed the left (or right) key when they did not feel the tactile sensation as L1–L2–L3. We recorded the response ratios and reaction times (RTs).

### Results and discussion

The response ratios of L1–L2–L3 tactile sensation and the RTs under each condition were averaged across participants (Figure 2, left panel). For the response ratios, a 3 (tactile patterns) × 2 (with vs. without flash) two-way ANOVA revealed a significant interaction between tactile pattern and the presence vs. absence of visual feedback, *F*(2, 22) = 6.65, *p* < 0.01. We also observed a simple main effect of flash under the L1–L1–L3 tactile condition, *F*(1, 33) = 17.12, *p* < 0.01, a simple main effect of tactile pattern under the flash condition, *F*(2, 44) = 65.47, *p* < 0.01, and a simple main effect of tactile pattern in the no-flash condition, *F*(2, 44) = 69.79, *p* < 0.01. These results were analyzed further using Ryan’s method of multiple comparisons (i.e., R-E-G-W’s *F* test). Under both flash conditions, each tactile pattern was significantly different from the others (*p*s < 0.05). Conversely, for RT, a similar two-way ANOVA with multiple comparisons conducted using Ryan’s method revealed only a significant main effect of tactile patterns, *F*(2, 22) = 7.28, *p* < 0.01. There were significant differences between L1–L2–L3 (average RT = 478.1 ms) and L1–L1–L3 (573.7 ms) and between L1–L2–L3 and L1-blank-L3 (587.2 ms; *p*s < 0.05) in terms of RT, indicating that regardless of the flash, participants more rapidly reacted to L1–L2–L3 stimuli.

Our results suggest that the tactile pattern of L1–L1–L3 could be felt as L1–L2–L3 to some extent. In other words, participants did experience the CRE (e.g., Blankenburg et al., 2006). Furthermore, a visual flash could enhance this illusion. Given that we presented the flash on L2 at *t*2, it seems likely that if the flash conveyed the location of tactile stimuli as L2 at *t*2, participants would feel the L1–L2–L3 tactile sensation instead of L1–L1–L3. However, the results of the L1-blank-L3 tactile condition suggest that the flash itself does not create a tactile sensation. This is because our tactile stimuli are sufficiently intense (cf., McKenzie et al., 2012). In sum, Experiment 1A suggests that as long as a flash is congruent with CRE saltation in terms of time and space, it can apparently relocate tactile stimuli to the location where the LED flashed; however, this multi-modal effect might not be reflected in participants’ RTs. The directional movement of a “cutaneous rabbit” indicates that a flash displaces tactile sensation in the direction of forward movement (forward displacement). In the following experiment, we examined whether a flash could also move the tactile location of the cutaneous rabbit backward, against its direction of saltation (backward displacement).

## Experiment 1B

Since the previous experiment suggested a cross-modal impact of forward displacement in the CRE, the current experiment examined the possibility of backward displacement. We predicted that as far as a visual flash is congruent with the CRE saltation (cross-modal correspondence), it should capture tactile location. A flash could also induce backward displacement just as easily as forward displacement. We expected that L1–L3–L3 tactile stimuli would also be felt as L1–L2–L3 to some extent. Furthermore, a flash on L2 at *t*2 was expected to introduce a stronger L1–L2–L3 tactile sensation. Conversely, if the direction of tactile displacement depended on the directional congruency of the whole tactile movement (forward or backward), L1–L3–L3 tactile stimuli with a flash would be felt as L1–L2–L3 to a lesser extent than L1–L1–L3 tactile stimuli with a flash.

### Method

Thirteen university students (1 male and 12 female, mean age = 19.9 years, range = 18–23) participated in a 3 × 2-factor experiment similar to Experiment 1A. We changed one tactile condition in this experiment. For the patterns of tactile stimuli, we presented three successive signals through vibrators: L1–L2–L3, L1–L1–L3, and L1–L3–L3 (only the last condition was replaced). Along with the same visual stimuli conditions used in Experiment 1A, we formed a sixth condition by presenting a one-shot signal on the LED on L2 at *t*2 (see Figure 2, right panel). These six conditions were randomly repeated 20 times for each participant. Participants judged whether the successive tactile sensation was L1–L2–L3, as in Experiment 1A.

### Results and discussion

For the response ratios, a 3 (tactile patterns) × 2 (with vs. without flash) two-way ANOVA revealed a significant interaction between tactile pattern and the presence vs. absence of visual feedback, *F*(2, 24) = 9.69, *p* < 0.01, a simple main effect of flash under the L1–L1–L3 tactile condition, *F*(1, 36) = 9.64, *p* < 0.01, a simple main effect of flash under the L1–L3–L3 tactile condition, *F*(1, 36) = 19.0, *p* < 0.01, a simple main effect of tactile condition in trials including a one-shot flash, *F*(2, 48) = 6.08, *p* < 0.01, and a simple main effect of tactile condition without a flash, *F*(2, 48) = 26.0, *p* < 0.01 (Figure 2, right panel). These results were analyzed further using Ryan’s method of multiple comparisons. In the no-flash conditions, each tactile pattern was significantly different from the others (*p*s < 0.05); further, the difference between the L1–L2–L3 and L1–L1–L3 conditions was not significant when flash was presented. For RT, a similar two-way ANOVA with multiple comparisons conducted using Ryan’s method revealed similar results to those in Experiment 1A: there was only a significant main effect of tactile pattern, *F*(2, 24) = 4.40, *p* < 0.05. There were significant differences in terms of RT between L1–L2–L3 (average RT = 642.6 ms) and L1–L1–L3 (739.8 ms) and between L1–L2–L3 and L1-blank-L3 (743.3 ms; *p*s < 0.05). This indicated that regardless of whether a flash was presented, participants most rapidly reacted to L1–L2–L3 stimuli.

This experiment replicated the result that the L1–L1–L3 tactile pattern could be felt as L1–L2–L3 and that this illusory sensation could be enhanced by a flash on L2 at *t*2. In this case, the sensation enhancement was almost the same as in the baseline condition (L1–L2–L3 tactile stimuli). It seems that participants made slightly more miss responses (approximately 10%) under the baseline condition in this experiment, while participants in Experiment 1A made hit responses almost perfectly. Since participants felt L1–L2–L3 tactile sensations under all conditions, they seem not to have been sufficiently conservative in their judgments. This might account for why RTs in the current experiment were approximately 150 ms longer than in the previous experiment. Furthermore, the L1–L3–L3 tactile pattern was also felt weakly compared with the L1–L2–L3 pattern. The CRE can occur when the second tactile sensation is subject to forward displacement (e.g., Blankenburg et al., 2006), indicating that stronger tactile displacement should be introduced when the direction of displacement is congruent with the direction of cutaneous rabbit saltation. This is apparently related to the fact that the human brain expects uniform motion and constant velocity of such motion, regardless of modality (Jones and Huang, 1982). This creates causality perception (Scholl and Tremoulet, 2000). Given that forward displacement is congruent with the direction of expected uniform motion, forward displacement in both unimodal (the CRE) and cross-modal (visual influence on the CRE) causality perception would be created more often (see the General Discussion). Nevertheless, the current experiment suggests that if a flash is given at an appropriate location and time, it can cause backward displacement. As a result, L1–L3–L3 tactile sensations enhanced by a flash yielded a similar perceived sensation to L1–L1–L3 tactility without a flash. However, the former sensations did not approach subjective similarity to the tactility of L1–L1–L3 stimuli enhanced by a flash. Experiments 1A and B collectively suggest that the CRE might be enhanced by congruent flashes relatively easily. In the following experiments, we examined whether or not the CRE could be attenuated by a spatially incongruent flash.

## Experiment 2A

The previous experiments suggested that a visual flash congruent with CRE saltation in terms of time and space would enhance the CRE, since this manipulation could lead participants to draw causal associations between visual and tactile sensation. This result might indicate that a visual flash can modulate the CRE; however, another possibility should also be examined. In the previous experiments, even if a flash simply modulated a single tactile location, the same results would be observed. To examine this possibility, we presented a spatially and temporally incongruent flash. This manipulation provides two possibilities. If a flash simply modulates a single tactile location, then the CRE should be attenuated in this setting; in other words, if a flash captures and relocates a tactile location to an incongruent location, then participants should feel less of an L1–L2–L3 sensation. On the contrary, if a flash modulates not a single tactile location but CRE saltation as a whole, we could expect that a spatially incongruent flash might not attenuate the CRE. This is because the cross-modal correspondence between the flash and CRE saltation should not be realized; as a result, the stimuli within each modality should be processed separately. We used the same tactile stimulus patterns as in Experiment 1B, but we delivered a flash on L1 at *t*2 in the current experiment.

### Method

**Figure 3:**
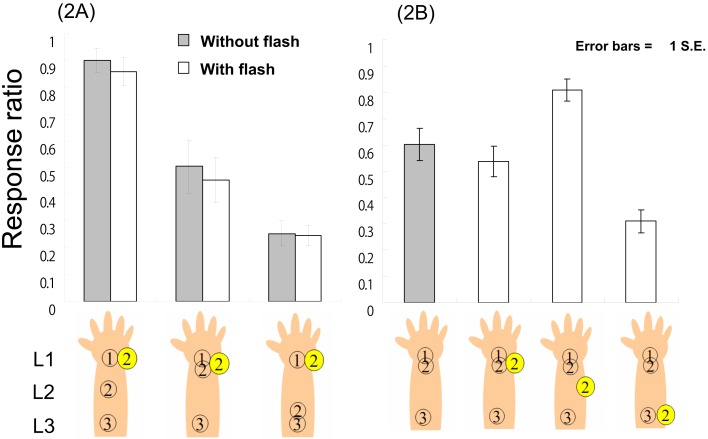
**The response ratio (L1–L2–L3 tactile feeling) in Experiment 2A and B**.

Thirteen university students (six male and seven female, mean age = 18.1 years, range = 18–19) participated in a 3 × 2-factor experiment similar to Experiments 1A and B. The tactile stimulus patterns were identical to those presented in Experiment 1B, but we changed the location of the flash in the current experiment: the one-shot signal from the LED was located on L1 and presented at *t*2 (see Figure 3, left panel). These six conditions were repeated 20 times for each participant, ordered randomly. The participants judged whether the successive tactile sensation was L1–L2–L3, as in Experiments 1A and B.

### Results and discussion

Using the response ratios as the dependent variable, a 3 (tactile patterns) × 2 (with vs. without flash) two-way ANOVA revealed no significant effects except for a significant main effect of tactile pattern, *F*(2, 24) = 20.37, *p* < 0.01. Multiple comparisons using Ryan’s method revealed that each tactile pattern was significantly different from every other pattern in terms of response ratio (*p*s < 0.05; Figure 3, left panel). Using RT as the dependent variable, a similar two-way ANOVA and multiple comparisons using Ryan’s method yielded the same results as Experiments 1A and B. That ANOVA revealed only a significant main effect of tactile pattern, *F*(2, 24) = 5.97, *p* < 0.01; further, there were also significant differences between L1–L2–L3 (average RT = 513.6 ms) and L1–L1–L3 (620.9 ms) and between L1–L2–L3 and L1-L3-L3 (623.4 ms; *p*s < 0.05) in terms of RT, indicating that regardless of the presence vs. absence of the flash, the participants reacted most rapidly to the L1–L2–L3 stimuli.

These results suggest that visual stimuli might not reduce the CRE though a flash on L1 at *t*2 must be spatially incongruent with the L1–L2–L3 tactile sensation. As for the L1–L2–L3 tactile conditions, if a flash captures and causes backward displacement of L2, participants would feel less of an L1–L2–L3 sensation. The fact that a flash did not reduce the response ratio indicates that it is difficult to observe simple spatial visuo-tactile ventriloquism (see General Discussion). The flash also did not reduce the response ratio in the L1–L1–L3 tactile conditions; even though, the flash was temporally and spatially synchronized with a second tactile stimulus (both on L1 at *t*2). Although the flash indicated the correct location of a second tactile stimulus, participants felt the CRE just as well as when no-flash was present. The effect of the flash also was not observed under the L1–L3–L3 tactile conditions. These results indicate that a spatially incongruent flash did not modulate the CRE. The tactile sensation seems to be processed separately from the visual system in the present experiment, indicating that a flash could modulate not a single tactile location but the CRE as a whole to the extent that the correspondence between the visual and tactile senses is maintained. To further examine the present results, we conducted Experiment 2B, wherein we fixed the tactile pattern as L1–L1–L3 and examined the effects of manipulating the flash location at *t*2.

## Experiment 2B

In order to examine the possibility that a flash could attenuate the CRE, we varied the flash location at *t*2 by fixing the tactile pattern as L1–L1–L3 (the typical CRE tactile pattern). While a spatially congruent L2 flash enhances the L1–L2–L3 tactile sensation (as we showed in Experiments 1A and B), L1 and L3 flashes are spatially incongruent with the CRE saltation. Although the results of Experiment 2A suggested that the L1 flash did not attenuate the CRE, there is a difference between L1 and L3 flashes: whereas an L1 flash might pull the illusory second tactile location (that is, L2) back to L1, the L3 flash might push it forward to L3. In the present experiment, congruency between displacement of the flash and the direction of CRE saltation was manipulated. As in Experiment 1B, we examined whether a flash at L3 could push the illusory L2 forward to L3 (i.e., the L1–L3–L3 sensation) and attenuate the CRE, where the correspondence between a flash and the CRE saltation might not be realized in this condition.

### Method

Fourteen university students (4 male and 10 female, mean age = 19.4 years, range = 18–21) participated in the current experiment. The tactile stimulus pattern was fixed as L1–L1–L3, and we varied the flash location at *t*2 (L1, L2, L3, or no-flash; see Figure 3, right panel). These four conditions were each repeated 20 times in random order for each participant. The judgment task was the same as in the previous experiments.

### Results and discussion

A one-way ANOVA using the response ratios as the dependent variable revealed a significant main effect of condition, *F*(3, 39) = 21,12, *p* < 0.01, and multiple comparisons (Ryan’s method) revealed that each tactile pattern was significantly different from the others (*p*s < 0.05), except for the difference between both with/without flash conditions on L1 (Figure 3, right panel). Similar analyses on RTs revealed a significant main effect of condition, *F*(3, 39) = 5.48, *p* < 0.01. Multiple comparisons revealed significant differences between the no-flash (average RT = 615.7 ms) and flash conditions (713.6 ms) on L3 and between the flash on L2 (590.2 ms) and flash-on-L3 conditions (*p*s < 0.05; average RT with flash on L1 = 678.8 ms).

These results essentially replicated the results of the previous experiments. A flash on L2 enhanced the CRE, but a flash on L1 did not attenuate the CRE as compared to the no-flash condition (in this case, L1–L1–L3 tactile stimuli; see the results of Experiments 1A and 2A). However, the newly added condition (a flash-on-L3) reduced the L1–L2–L3 tactile sensation; further, this condition generated the longest RTs among the four conditions. One possible reason for the differences between the L1 and L3 flash conditions (both are spatially incongruent) is the factor of congruency with the direction of CRE saltation. As we suggested in Experiment 1B, forward displacement should be easier than backward displacement in the CRE; however, enhancement of the L1–L2–L3 tactile sensation by a flash could occur even under conditions favorable to backward displacement. However, that type of attenuation was not observed in the current experiment, indicating that the cross-modal correspondence problem and the direction of visual displacement of the tactile location interact. When cross-modal correspondence (spatial and temporal congruency between the flash and CRE saltation) is present, the flash causes forward or backward displacement. As a result, the CRE is enhanced (Experiment 1A and B). Conversely, when cross-modal correspondence is not provided, forward instead of backward displacement is created (Experiment 2A), attenuating the CRE (the current experiment). To investigate why the CRE could not be attenuated under the flash conditions favoring backward displacement, we required participants to report the tactile sensations as they were felt instead of implementing a two forced-choice response task (as in Experiment 2B).

## Experiment 2C

We required participants to report their tactile sensation as it was experienced in order to examine why the attenuation of the L1–L2–L3 tactile sensation by a flash might not be observed in some conditions. We hypothesized that when a flash does not correspond to CRE saltation, it might affect a single concurrent tactile stimulus. Furthermore, since forward displacement is relatively easily realized, as suggested by the results of Experiment 1B, it was expected that only forward displacement would emerge to attenuate the CRE. In the current experiment, the tactile stimuli were identical to those used in Experiment 2A, and a flash was presented on either L1 or L3 (both are spatially incongruent with the CRE saltation). We examined what participants felt when they did not feel the L1–L2–L3 tactile sensation under conditions favoring forward or backward displacement.

### Method

**Figure 4:**
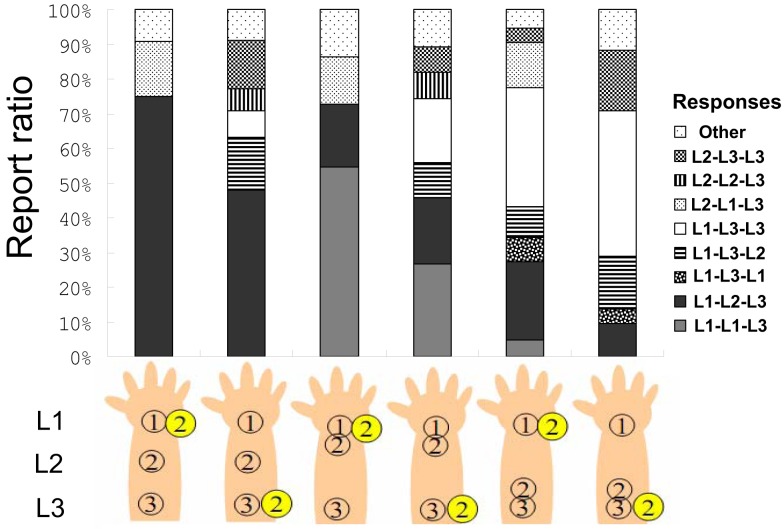
**The report ratio in Experiment 2C**.

In the current experiment, because more participants were required in order to achieve stability in the self-reporting of tactile sensation (see below), the number of participants was increased. This was done in order to account for the several potential variations in responses. Sixteen university students (4 male and 12 female, mean age = 19.8 years, range = 18–24) participated in a 3 × 2 factorial experiment: the tactile stimulus patterns were L1–L2–L3, L1–L1–L3, or L1–L3–L3, and a flash was presented at *t*2 on L1 or L3 (see Figure 4). These six conditions were repeated 20 times in random order for each participant. Participants were required to report the pattern of tactile stimuli corresponding with their sensation using a key press as they felt it. Since they were informed before the experiment that they would feel three successive tactile stimuli, they reported three locations in order without time constraints (this was not the case in the previous experiments.)

### Results and discussion

We summarized the participants’ raw reports. Figure 4 shows the tactile patterns reported by the participants after we omitted rare responses (below 4%) and labeled them as “other” responses in each condition. The report ratios of L1–L2–L3, L1–L1–L3, and L1–L3–L3 comprised almost 70% of all responses. A 3 (tactile pattern) × 2 (flash location) two-way ANOVA using the report ratio of L1–L2–L3 as the dependent variable revealed a significant interaction between tactile pattern and flash location *F*(2, 30) = 9,17, *p* < 0.01, significant simple main effects of flash condition under the L1–L2–L3 and L1–L3–L3 tactile conditions (*p*s < 0.01), and significant simple main effects of tactile pattern under both flash conditions (*p*s < 0.01). Multiple comparisons using Ryan’s method revealed that the report ratio of the L1–L2–L3 tactile pattern was significantly different from those of the L1–L1–L3 and L1–L3–L3 patterns under both flash conditions (*p*s < 0.01).

First, we need to consider the potential effects of the requirements placed on participants. In the current experiment, participants reported the tactile sensation pattern they felt. Compared with the results of Experiments 2A and B, the report ratios for the L1–L2–L3 sensation were generally reduced. This might be because participants were placed under time pressure during the previous experiments; thus, they might have encoded other potential pattern identifications, like L2–L1–L3 or L1–L3–L2, as L1–L2–L3. That might also explain the lack of significance of flash condition under the L1–L1–L3 tactile conditions. It seems that the feeling of the L2–L1–L3 pattern could be judged as L1–L2–L3 when responses are made quickly (e.g., Experiments 2A and B; see Figure 4). The sum of the response ratios of L1–L2–L3 and L2–L1–L3 under the L1 flash condition in the current experiment approximates the response ratio of L1–L2–L3 under the same conditions in Experiments 2A and B. That notwithstanding, why were reports of feeling the L2–L1–L3 or L1–L3–L2 patterns observed (each at a 10–15% rate) in the current experiment? Furthermore, why were these reports encoded as L1–L2–L3 tactile responses in the previous experiments? We discuss these questions in terms of the temporal order judgment between vision and tactile perception in the General Discussion.

Although the presentation of a flash on L1 or L3 is supposed to be spatially incongruent with the tactile feeling of L1–L2–L3, the significant differences between these presentations signify the importance of congruency with the direction of cutaneous rabbit saltation. The attenuation of the CRE might occur only under conditions of forward displacement. Under these conditions, we observe more reports of L1–L3–L3 or L2–L3–L3, which are congruent with the direction of the CRE, instead of L1–L2–L3. The CRE is affected by directional congruency between tactile displacement and the whole tactile movement (i.e., the comparison between the L1–L1–L3 and L1–L3–L3 tactile conditions, as suggested by the results of Experiment 1B). This is also true when the flash modulates the tactile location. The flash caused forward displacement (attenuating the CRE) but not backward displacement (no attenuation of the CRE) in Experiment 2. On the other hand, the flash caused both forward and reverse displacement (both enhancing the CRE) in Experiment 1. Although the reason for this asymmetry in the ease of enhancement vs. attenuation is still unclear, the brain might intrinsically expect consistently moving tactile sensations, such as something hopping along the skin (Goldreich, 2007) or off of the body (Miyazaki et al., 2010). Our somatosensation might be specialized for detecting other species creeping or hopping along our skin; thus, we might prefer false alarms to misses, since the ability to perceive external agents in motion is strongly ingrained (Leslie, 1995; Schultz et al., 2005; Pratt et al., 2010).

## General Discussion

Our present results suggest that a simple flash is able to modulate the CRE. Congruency between the direction of tactile displacement (forward or backward displacement) and CRE saltation and the existence of cross-modal correspondence between visual and tactile cues seem to play key roles in this ability. First, Experiment 1B suggested that the CRE itself is susceptible to modulation by movement direction, indicating that forward displacement would be more acceptable than backward displacement in the CRE. The results of Experiments 1A and B suggest that when a flash is temporally and spatially congruent with the CRE (when a flash is placed at the location of the supposed CRE saltation), and cross-modal correspondence is provided, participants feel the CRE robustly regardless of the direction of tactile displacement. Conversely, the results of Experiments 2A, B, and C suggest that when a flash is spatially incongruent with the CRE saltation, and cross-modal correspondence is not provided, participants experience an attenuated CRE under conditions favoring forward displacement, but not under conditions favoring backward displacement. The results of Experiment 2C also indicate that the combination of these two factors creates various tactile sensations and that temporal order judgment might be modulated under some conditions. Our results provide theoretically important information on unimodal and multi-modal causality perception: the former indicates that our brain needs to detect motion from successive stimuli, and the latter indicates that multi-modal presentation creates a unified, cross-modal representation of motion. Though a previous study suggested that the CRE is the spatiotemporal dynamics of an early, unimodal, sensory map (Flach and Haggard, 2006), the CRE is related not only to a lower level of spatiotemporal perceptual interaction but also a higher level of cognition that includes unimodal causality or animacy perception (Scholl and Tremoulet, 2000). The present study suggests that forward displacement (i.e., constant movement in one direction) of tactile sensation is more acceptable than backward displacement, in accordance with the constant velocity assumption (Jones and Huang, 1982; see the following section). The current study also suggests, for the first time, that unimodal causality perception (the CRE) might be influenced by multi-modal presentation under conditions of cross-modal causality correspondence (Schutz and Kubovy, 2009; Parise et al., 2012).

While one previous study argued that the CRE is not affected by concurrent auditory temporal information (Flach and Haggard, 2006), the current study suggests that the CRE is affected by a concurrent visual flash, where “spatiotemporal” synchronicity among modalities is provided. The spatiotemporal dynamics of a somatosensory map in the CRE (Flach and Haggard, 2006) might only be modulated by spatiotemporally synchronized stimuli from other modalities. We discuss these specific mechanisms below.

### Somatosensory system in terms of time and space

The somatosensory system, as well as other modalities, faces not only spatial but also temporal imprecision. The most discriminating tactile sensors among primates – the fingertips – house a few hundred sensory nerve fibers per square cm (Johansson and Vallbo, 1979; Darian-Smith and Kenins, 1980), a density four orders of magnitude below the peak density of retinal ganglion cells (Wassle et al., 1990). Nevertheless, the spatial attributes of tactile sensory nerves are likely important. The brain possesses a somatotopic body map within the primary somatosensory cortex (S1; Penfield and Boldrey, 1937) to reflect the locations of physical stimuli on the skin. Furthermore, whereas sound stimuli may provide temporal precision to the perception of spatial attributes (Kubovy and Van Valkenburg, 2001), tactile stimuli may be less temporally defined (Keetels and Vroomen, 2008a,b). Though the CRE might run contrary to the spatial tactile attributes mentioned above, this illusion reflects not only the temporal and spatial imprecision of the somatosensory system but also the brain’s expectations regarding the external world (Goldreich, 2007). Previous studies have suggested that the human brain expects uniform motion, regardless of modality (Jones and Huang, 1982). Sensory systems work to recover the causal and social structures of the world by inferring properties such as causality and animacy (Scholl and Tremoulet, 2000). In line with this assumption, the CRE, tau effect, and kappa effect share the same basis: the constant velocity assumption (Jones and Huang, 1982).

A typical tau effect, where the perceived distance between stimuli underestimates, and grows in proportional with, the actual distance when stimulus timing is held constant (Marks et al., 1982; Cholewiak, 1999) and the kappa effect, in which the perceived time between stimuli dilates as the distance between stimuli is increased (Suto, 1952), reflect just two fundamental perceptual distortions: underestimation of inter-stimulus distance (perceptual length contraction) and overestimation of inter-stimulus time interval (perceptual time dilation; Goldreich, 2007). Given that three successive tactile stimuli define two spatial (S1 and S2) and two temporal intervals (T1 and T2), the somatosensory system intuitively imputes motion at a given speed to the tactiles and tries to equalize the ratios S1/S2 and T1/T2; thus, it follows that S1/T1 = S2/T2 (modified from Jones and Huang, 1982). In this way, the sensory system – which includes somatosensation, vision, and audition (Cohen et al., 1953; Shore et al., 1998) – attempts to equalize the velocity between the first and second stimuli (S1/T1) and that between the second and third stimuli (S2/T2); this is known as the constant velocity assumption (Goldreich, 2007). Though the CRE is also in line with the tau and kappa effects, the present study also suggests that the direction of tactile displacement is crucial. The L1–L1–L3 tactile stimuli are felt more as L1–L2–L3 than as L1–L3–L3 (Experiment 1B). The constant velocity assumption is rooted in the notion that motion perception is closely related to animacy perception or detection of the movement of external agents (Leslie, 1995; Scholl and Tremoulet, 2000). Thus, it is reasonable that not only the speed but also the direction of motion should be equalized for motion perception. Because forward displacement is congruent with the expected direction of uniform motion, the brain might perceptually relocate illusory sensations in the forward direction, as changes in the motion signal usually create forward displacement of representational momentum. Thus, the general tendency is to displace the judged position of a moving target as being relatively far forward along the path of motion (Tremoulet and Feldman, 2006; Getzmann and Lewald, 2007, 2009).

### Visuo-tactile interaction in terms of cross-modal correspondence

When sensory signals are presented simultaneously across multiple modalities, they tend to be detected more quickly, accurately, and at lower thresholds than if the same signals are presented individually (e.g., Hershenson, 1962; Frassinetti et al., 2002). In addition, if those signals are incongruent, various multi-modal illusions will be observed as far as the temporal synchronicity among modalities is provided. The McGurk effect is a perceptual phenomenon that demonstrates interactions between hearing and vision in speech perception (McGurk and MacDonald, 1976). This effect can be experienced when the visual representation of a phoneme is dubbed with a sound recording of a different phoneme being spoken; in such situations, the perceived phoneme is often a third, intermediate phoneme. Moreover, spatial ventriloquism occurs when the visual locations of stimuli capture and displace their auditory locations (Jack and Thurlow, 1973; Alais and Burr, 2004). Certain visuo-tactile interactions have also been reported. When participants discriminate the locations of vibrotactile stimuli by ignoring distractor lights, such tactile discriminations are slowed when the distractor light is incongruent with the tactile target (Pavani et al., 2000). In addition, the perceived number of tactile stimuli is influenced by the number of flashes presented (and vice versa; Violentyev et al., 2005; Bresciani et al., 2006); there have also been reports of bidirectional attentional blink between vision and touch (Soto-Faraco et al., 2002).

Vision also captures tactile sensations. The rubber hand illusion (RHI) refers to the effect of watching a rubber hand being stroked synchronously with one’s own, unseen hand. Viewing this for a short time causes the observer to perceptually assimilate the rubber hand into his or her own body (Botvinick and Cohen, 1998). The RHI might indicate that the visual location of stimuli displaces the tactile one [e.g., a typical subjective feeling for RHI is, “It seemed as if I were feeling the touch of the paintbrush… where I saw the rubber hand (being) touched” (Asai et al., 2011; Botvinick and Cohen, 1998)]. In addition, aspects of the sense of body ownership such as body posture (Austen et al., 2004; Ehrsson et al., 2004), visual appearance (Tsakiris and Haggard, 2005), hand identity (Tsakiris et al., 2006), or the self-other representation (Schutz-Bosbach et al., 2006; Asai et al., 2011) also strongly affect the RHI. To our knowledge, so-called simple “visuo-tactile spatial ventriloquism” cannot be observed (see also our results of Experiment 1A): we do not simply feel tactile sensations on locations where the light flashes, except when we are attempting to detect near-threshold signals (i.e., light-evoked false alarms; McKenzie et al., 2012). Though the concept has still not been completely elucidated, the somatotopy of tactile body location in the brain might be responsible for these results (cf., the tonotopy of audition). Thus, temporal ventriloquism has been reported in the domain of visuo-tactile interaction, suggesting that small amounts of latency between vision and touch are reduced and tend to go unnoticed (Spence et al., 2001; Keetels and Vroomen, 2008a; Vroomen and Keetels, 2010).

Although previous studies have not reported such spatial visuo-tactile ventriloquism, our results suggest that we feel the tactile sensation where the light flashes in several specific situations. This suggests that although a flash might not relocate a tactile stimulus, a flash could modulate illusorily located tactile sensation, as visual influences on the tau and kappa effects have been reported (Suto, 1952; Russo and Dellantonio, 1989). Cross-modal causality plays a key role in governing the integration of sensory information, depending on its ecological plausibility (Schutz and Kubovy, 2009). Humans can use the similarities between the temporal structures of sensory signals in different modalities to solve the correspondence problem, ultimately inferring causation from correlation (Parise et al., 2012). Given that people infer which signals have common underlying causes and hence merit integration (Parise et al., 2012), the most common perceived cause in the current study – in which we observed visuo-tactile integration (cross-modal causality) while focusing on the CRE (unimodal causality) – ought to be the external agent in motion (Leslie, 1995; Scholl and Tremoulet, 2000).

### Temporal order judgment and cross-modal influence

However, there might not be any differences between the actual and illusorily located tactile sensations within the human brain. Illusory sequences activate the contralateral primary somatosensory cortex at somatotopic locations corresponding to the filled-in illusory perceptions on the forearm (Blankenburg et al., 2006); this suggests that this illusion is associated with the early sensory body map represented in S1. Why does the cutaneous rabbit hop toward the light, and why is the CRE attenuated, even though illusorily located tactility should be represented within a corresponding area of S1? One possible reason might be related to temporal visuo-tactile ventriloquism. Small amounts of latency between vision and touch (or sound) tend to be reduced and go unnoticed, because the timing of visual events is flexible and adjusts immediately (for a review, see Vroomen and Keetels, 2010). The results of Experiment 2C indicated that the pattern of tactile feeling could often be predicted by the flash. Participants’ report ratios of L2–L1–L3 (under L1 flash conditions, 10–15%) and L1–L3–L2 (under L3 flash conditions, 10–15%) are of interest. The former might be interpreted as reflecting the participants’ inclination toward answering L1–L2–L3 (i.e., the CRE), and the L1 flash might rearrange the temporal order into L2–L1–L3. The latter is also as well. The flash might not modulate the extent to which the second tactile is felt in S1 but does modulate the temporal order of the tactile sequence. As a result, a flash could attenuate the CRE, especially when participants have enough time to report their sensation. The CRE and its interaction with vision indicate that sensory events at a given time point are influenced by future sensory events; this is called “postdictive” sensation (Eagleman and Sejnowski, 2000).

If we accept this two-step explanation of the visuo-tactile interaction, other interpretations regarding the lack of CRE attenuation caused by the L1 flash (Experiment 2) might be possible. The current results indicate that a flash captures the tactile location, except when the flash is presented on L1. However, even if a flash on L1 captured the tactile location, it would not change the current results. If we feel the sensation of the flash location, the L1–L2–L3 tactile stimuli with flash on L1 at *t*2 might be felt as L1–L1–L3. This illusory L1–L1–L3 tactile sensation could be re-encoded as L1–L2–L3, according to the CRE. One might expect longer RTs under the flash than the no-flash conditions, but we did not observe this pattern of results (see Experiment 2A). Although no clear conclusions can be drawn regarding the influence of the presentation of a flash on L1, the results indicate that it modulates successive tactile sensations spatially, temporally, or both.

### Limitations of the current study

The current paradigm has some limitations, and further research is needed to expand our knowledge on the effects of visual flash on the CRE. First, visual capture of the CRE might be susceptible to response bias in Experiments 1 and 2. For instance, previous studies examining the CRE required participants to judge whether or not the tactile stimuli were presented on L2 (e.g., Blankenburg et al., 2006), not to judge whether or not the tactile stimuli were felt as L1–L2–L3 (as in the current study). Because we examined multi-modal interaction, the temporally congruent feeling of tactility on L2 does not always equal an L1–L2–L3 sensation. This can be inferred from the results of Experiment 2C, in which participants reported feeling various patterns of tactile stimuli, including tactile sensation on L2. Since the results of Experiments 2A and B suggested that participants did not simply follow the flash, we are optimistic about this possibility. Furthermore, the current study did not consider participants’ attention in detail: we simply instructed participants to see their left arm as a whole, since the CRE is not affected by gaze direction (Flach and Haggard, 2006). Nevertheless, potential visual effects of attention on tactility (Pavani et al., 2000; Soto-Faraco et al., 2002) should be controlled in future studies. Finally, it is possible that participants felt more than three tactile sensations because of the flash (Bresciani et al., 2006); however, we could not directly test this possibility, given that we informed participants that they would experience three successive stimuli. Although no participant reported such sensations, this question is worth examining.

## Conclusion

The CRE is an attractive phenomenon, as postdictive processing is one of the key concepts that characterizes our conscious perception (Miyazaki et al., 2010). However, this mechanism includes temporal and spatial factors that are difficult to ascertain (Flach and Haggard, 2006; Goldreich, 2007). While the CRE might have the spatiotemporal dynamics of an early, unimodal, sensory map (Blankenburg et al., 2006; Flach and Haggard, 2006), other studies have also suggested that attention or expectations (e.g., Kilgard and Merzenich, 1995), body posture (Eimer et al., 2005), and extended body schemas (Miyazaki et al., 2010) would affect the CRE. For the first time, our results suggest that a simple visual flash could modulate the CRE. Experiment 1 suggested that the CRE itself is susceptible to movement direction and that forward displacement within the CRE would be more acceptable. Furthermore, when a flash is temporally and spatially congruent with CRE saltation (i.e., cross-modal correspondence is provided), participants feel the CRE more robustly regardless of the direction of tactile displacement. Conversely, Experiment 2 suggested that when a flash is spatially incongruent with CRE saltation (i.e., cross-modal correspondence is not provided), participants feel the CRE to a lesser extent under conditions favoring forward than backward displacement. Participants’ raw reports also indicated that the combination of these two factors creates various tactile sensations and that temporal order judgment is modulated under some conditions. Our results reflect (1) how the human brain interprets successive stimuli in terms of time and space (i.e., motion or causality perception according to the constant velocity assumption) and (2) that available information from other modalities provides key clues (cross-modal enhancement/attenuation of unimodal causality) about the extent to which the modalities are spatiotemporally synchronized (i.e., cross-modal correspondence). We suggest that the CRE needs to be considered not only as a perceptual phenomenon, but also as a higher cognitive function including spatiotemporal causality or animacy inferences in both unimodal and multi-modal domains.

## Conflict of Interest Statement

The authors declare that the research was conducted in the absence of any commercial or financial relationships that could be construed as a potential conflict of interest.
